# A Socio-Demographic Examination of Adults Responding to Governmental Vaccination Recommendations during the Japanese Rubella Outbreak of 2013

**DOI:** 10.1371/journal.pone.0129900

**Published:** 2015-06-09

**Authors:** Ai Hori, Koji Wada, Derek R. Smith

**Affiliations:** 1 Department of Epidemiology and Prevention, Clinical Research Center, National Institute for Global Health and Medicine, Tokyo, Japan; 2 Bureau of International Health Cooperation, National Institute for Global Health and Medicine, Tokyo, Japan; 3 School of Health Sciences, Faculty of Health and Medicine, University of Newcastle, Ourimbah, Australia; Hamamatsu University School of Medicine, JAPAN

## Abstract

**Background:**

In 2013 a rubella outbreak occurred among Japanese people of working-age which resulted in 14,357 reported cases. The Japanese government subsequently recommended voluntary vaccination or rubella antibody testing for young women (15–49 years of age) who were planning to conceive and for adult men, children, and other persons in potential contact with pregnant women at home. However, the expense and time involved for vaccination, antibody testing and visiting a clinic may represent a major barrier to voluntary compliance among this busy demographic. The aim of the current study was, therefore, to examine potential relationships between the social background of Japanese working-age individuals affected by the 2013 voluntary vaccination campaign.

**Methods:**

A web-based survey of 1,889 Japanese men and women aged 20–49 years was conducted in early 2014. Statistical analyses were used to explore the associations between social background and testing for rubella antibody and / or vaccination uptake during the previous year.

**Results:**

Twenty-four percent of respondents who were planning a pregnancy had been tested for rubella antibody or vaccinated in 2013. However, among those without a current desire for pregnancy, 3% of men and 7% of women, respectively, were tested or vaccinated. Regardless of whether they were planning to conceive, testing for rubella antibodies or vaccination was statistically associated with having acquaintances who had been vaccinated, understanding the government recommendations, and being able to confirm their lack of rubella vaccination history using Maternal and Child Health Handbook records in both men and women.

**Conclusion:**

To help eliminate rubella in Japan, additional initiatives need to target Japanese individuals who cannot envisage a direct benefit from vaccination. The results of this study suggest that disseminating the government recommendation to all potentially affected subpopulations, along with maintaining life-time vaccination records might offer a solution to encourage vaccination uptake among working-age adults in Japan, as elsewhere.

## Introduction

In 2013, a rubella outbreak occurred among the working-age population in Japan [1], from which local authorities reported a total of 14,357 rubella cases to the federal government [2]. In compliance with the Act on Prevention of Infectious Diseases and Medical Care for Patients Suffering Infectious Diseases, up to 40 cases of congenital rubella syndrome [3] were also reported. Ninety percent of the reported cases in the 2013 rubella outbreak were adults who had not been previously vaccinated or whose vaccination histories were unknown. Over 60% of the reported cases were among men aged 20–49 years. Booster vaccinations of rubella-containing vaccine had not routinely been given in Japan until 2006, and Japanese men born before April 1979 had not been vaccinated as children [4].

The Japanese Ministry of Health, Labour and Welfare has recommended that specific groups receive rubella vaccination or an antibody test for rubella, and to then be vaccinated if low immunity is clinically indicated. These groups are: 1) women in their late teens to 40s, particularly those who intend to conceive or who have a high chance of becoming pregnant; 2) individuals associating with pregnant women, such as husbands, children, and other family members who reside with these women; and 3) women in the early postpartum period, except where an adequate antibody titer has been confirmed [5–6]. Public health evidence indicates that vaccination coverage rates should be rapidly increased to effectively eliminate rubella in populations [7–8]. Medical societies including the Japan Society of Obstetrics and Gynecology (Tokyo) [9] and the Japan Pediatric Society (Tokyo) [10] have released public statements that encourage all adults, but especially men, to be vaccinated so that the goal of eliminating rubella can be achieved.

The proportion of individuals who responded to the 2013 campaign to encourage voluntary rubella vaccination is unknown, while the social backgrounds of these individuals are also unknown. In 2014, the Japanese government set a goal to eliminate rubella nationwide by 2020 [11]. However, an additional public education campaign may also be necessary to increase vaccination uptake, especially for adults. The aim of the current study was, therefore, to examine potential relationships between the social background of Japanese working-age individuals affected by the 2013 voluntary vaccination campaign.

## Materials and Methods

### Data collection

A total of 1,889 Japanese adults, aged 20–49 years, were recruited for this study using a web-based survey company registry. In early January 2014, the survey company randomly selected 3,423 persons from a list of 1.8 million individuals and invited them to participate in the study. Those who agreed to participate were subsequently directed to complete an anonymous online questionnaire. Registrants were provided financial incentives for their participation. Recruitment was ended once the number of participants had reached approximately 1,800 individuals.

### Questionnaire

The survey questions included basic demographic information such as; age, gender, marital status (single, married, or divorced), and highest education level completed (less than high school, college or vocational school, or university or greater). With regard to a current desire for pregnancy (either partner or self), each participant was asked “Do you or your spouse (partner) currently have a desire to become pregnant? (Yes/No)”. To determine whether a respondent’s acquaintances were vaccinated against rubella in 2013, each participant was asked “How many of your adult acquaintances (children excluded) received a rubella vaccination between January 1 and November 30 in 2013?”. The responses were categorized as none (No) or one or more (Yes) in the analysis. To determine a respondent’s understanding of the government recommendation regarding rubella vaccination, we then asked, “Are you aware that people associating with pregnant women, such as husbands, children, and other family members who live with these women, and women who are in their late teens to 40s (particularly those who desire to conceive a baby or who have a high chance of becoming pregnant) are recommended to receive a rubella vaccination, so that pregnant women will be protected, except when these people have a confirmed adequate antibody titer? (Yes/No)”. To determine whether an individual’s rubella vaccination history could be confirmed, we asked, “At present, did you have access to your records such as the Maternal and Child Health Handbook (not including your parents’ recollection) to confirm whether or not you have previously been vaccinated against rubella? (Yes/No)”. Participants were also asked the following questions to identify their perceptions of the risks associated with the rubella outbreak: “Are you aware that a rubella has been occurring this year, particularly among adults? (Yes/No)”, “Are you aware that babies may develop a serious condition, congenital rubella syndrome, when carried by mothers who are infected with rubella? (Yes/ No)”, and “Do you know that this year’s epidemic of rubella among adults has caused an increase in the number of infants who have the aforementioned serious disease called congenital rubella syndrome? (Yes/No)”.

To determine a respondent’s rubella testing and vaccination history between January 1 and November 30, 2013, we asked “Did you receive an antibody test for rubella? (Yes/No)” and “Did you get a rubella vaccination? (Yes/No)”. We combined the answers to these questions in the analysis to test a categorical outcome variable (tested for antibody or vaccinated against rubella).

### Statistical analysis

We performed a univariate analysis using Pearson’s chi-square test to examine potential relationships between receiving vaccination and a current plan to become pregnant (either themselves or their partner). Binomial logistic regression analyses were then used to explore the possible associations between social background and the history of testing for rubella antibody or being vaccinated during the January to November 2013 period. Multivariate analyses were performed to adjust for age (continuous) and education level. Binomial logistic regression was then performed to explore the associations between social background, desire for pregnancy (either themselves or their partner) and their history of testing for rubella antibody or being vaccinated. IBM SPSS Statistics 19 software (IBM Corporation, Armonk, NY, USA) was used for all analyses. A result was considered to be statistically significant at p-values <0.05. Given that the values for outcome proportions were relatively high, Zhang’s formula was used to adjust the odds ratios for common outcomes, as previously described [12].

### Ethics statement

This study was approved by the Human Research Committee at National Center of Global Health and Medicine (Tokyo, Japan).

## Results

A total of 1,889 persons (945 men and 944 women) participated in the study. Table 1 indicates the demographic characteristics of the participants. Almost one quarter (22.4%) reported a current desire pregnancy for their partner or themselves. Almost 10% (9.3%) of participants had been tested for rubella antibodies or had been vaccinated against rubella in 2013. The Japanese government recommendation that adults receive a vaccination against rubella was understood by 777 (41.1%) of the respondents.

**Table 1:** Participant characteristics (N = 1,889).

		n	(%)
**Age (y)**			
	20–29	632	(33.5)
	30–39	627	(33.2)
	40–49	630	(33.4)
**Gender**			
	Men	945	(50.2)
**Education completed**			
	Less than high school	510	(27.0)
	College or vocational school	471	(24.9)
	University or greater	878	(46.5)
**Current desire for pregnancy for partner or herself**			
	Yes	423	(22.4)
**Tested rubella antibody levels in 2013**			
	Yes	120	(6.4)
**Vaccinated against rubella in 2013**			
	Yes	99	(5.2)
**Tested rubella antibody levels or vaccinated against rubella**			
	Yes	175	(9.3)
**Acquaintance who have had the rubella vaccination**			
	More than one	90	(4.8)
**Understand the government recommendation**			
	Yes	777	(41.1)
**Being able to confirm a rubella vaccination history by the Maternal and Child Health Handbook**			
	Yes	688	(36.4)
**Marital status**			
	Single	899	(47.6)
	Married	916	(48.5)
	Divorced	74	(3.9)
**Awareness of the rubella outbreak among adults in 2013**			
	Yes	1155	(61.1)
**Awareness of the possibilities of congenital rubella syndrome which can occur in cases of rubella infection during pregnancy**			
	Yes	1291	(68.3)
**Awareness of the increasing number of congenital rubella syndrome cases**			
	Yes	659	(34.9)

Table 2 presents the results comparing the proportion of persons who were tested for antibody or had been vaccinated against rubella versus their current desire to conceive. Among those who were planning to conceive, 24.3% of men and 24.4% of women had been tested for rubella antibodies or vaccinated against rubella in 2013. By contrast, 2.9% of the men and 7.3% of the women without a current desire for pregnancy had undergone antibody testing or had been vaccinated. Results demonstrated a significant difference in the proportion who had undergone antibody testing or had been vaccinated versus current desire for pregnancy (p<0.01).

**Table 2:** Participants who had undergone antibody testing or had been vaccinated against rubella during the 2013 outbreak in Japan.

	Men				Women			
	Current desire for pregnancy for partner				Current desire for pregnancy for themselves			
	Yes		No		Yes		No	
	(n = 148)		(n = 797)		(n = 225)		(n = 669)	
	n	(%, 95%CI)	n	(%, 95%CI)	n	(%, 95%CI)	n	(%, 95%CI)
**Tested rubella antibody in 2013**	24	(16.2, 10.3–22.2)	5	(0.6, 0.2–1.5)	53	(19.3, 14.6–23.9)	38	(5.7, 3.9–7.4)
**Vaccinated rubella in 2013**	31	(20.9,14.4–27.5)	20	(2.5,1.4–3.6)	29	(10.5, 6.9–14.2)	19	(2.8,1.6–4.1)
**Tested rubella antibody levels or vaccinated against rubella**	36	(24.3,17.4–31.2)	23	(2.9,1.7–4.0)	67	(24.4, 19.3–29.2)	49	(7.3, 5.4–9.3)

Table 3 indicates the statistical associations between antibody testing or vaccination against rubella, and the study variables. Current desire for pregnancy (either partner, spouse, or themselves [for women]) was associated with testing rubella antibody levels or being vaccinated against rubella for men (Odds Ratio [OR]: 3.31; 95% Confidence Interval [CI]: 1.94–5.08) and women (OR: 1.80; 95% CI: 1.19–2.64).

**Table 3:** Logistic regression analysis regarding vaccination or antibody testing for rubella during 2013 in Japan (Men and Women).

	Men		Women	
	Adjusted	95%CI	Adjusted	95%CI
**Currently planning to conceive (partner, spouse or themselves)**	3.31	(1.94–5.08)	1.80	(1.19–2.64)
**Acquaintances who have been vaccinated for rubella**	4.69	(2.59–7.03)	2.80	(1.64–4.39)
**Understand the government’s recommendation**	4.02	(2.09–6.41)	3.09	(1.70–4.97)
**Being able to confirm rubella vaccination history by the Maternal and Child Health Handbook**	2.76	(1.57–4.41)	2.10	(1.37–3.06)
**Marital Status**				
** Single**	ref		ref	
** Married**	1.71	(0.85–3.19)	3.03	(1.83–4.62)
** Divorced**	1.80	(0.23–7.01)	1.32	(0.27–4.61)
**Education completed**				
** Less than high school**	1.01	(0.52–2.12)	0.56	(0.31–1.06)
** College or vocational school**	0.56	(0.21–1.41)	0.95	(0.57–1.55)
** University or greater**	ref		ref	
**Awareness of the rubella epidemic among adults in 2013**	0.73	(0.30–1.67)	1.59	(0.67–3.36)
**Awareness of the possibilities of congenital rubella syndrome which occurs in cases of rubella infection during pregnancy**	1.52	(0.56–3.57)	1.10	(0.30–3.32)
**Awareness of the increasing number of congenital rubella syndrome cases**	0.78	(0.38–1.54)	1.67	(1.04–2.58)

CI: Confidence Interval; ref: referent

Tables 4 and 5 present the statistical associations between antibody testing or vaccination against rubella, and the study variables by current desire to conceive among either one’s spouse or themselves. Of those with a current desire for pregnancy, 61 (41.2%) men and 99 (36.0%) women did not understand the government’s rubella recommendations.

**Table 4:** Vaccination or antibody testing for rubella in 2013 for men.

	Current desire for pregnancy for partner				Without current desire for pregnancy for partner			
	Vaccinated or tested		Not vaccinated or tested		Vaccinated or tested		Not vaccinated or tested	
	(n = 36)		(n = 112)		(n = 23)		(n = 774)	
**Having acquaintances who have had rubella vaccination**								
** **Yes	13	(65.0)	7	(35.0)	5	(27.8)	13	(72.2)
** **No	23	(18.0)	105	(82.0)	18	(2.3)	761	(97.7)
**Understand the government’s recommendation**								
** **Yes	32	(36.8)	55	(63.2)	16	(8.0)	184	(92.0)
** **No	4	(6.6)	57	(93.4)	7	(1.2)	590	(98.8)
**Being able to confirm rubella vaccination history by the Maternal and Child Health**								
** **Yes	28	(43.8)	36	(56.2)	14	(7.1)	183	(92.9)
** **No	8	(9.5)	76	(90.5)	9	(1.5)	591	(98.5)
**Marital Status**								
** **Single	10	(23.3)	33	(76.7)	9	(1.8)	497	(98.2)
** **Married	26	(25.5)	76	(74.5)	13	(4.9)	252	(95.1)
** **Divorced	0	(0)	3	(100)	1	(3.8)	25	(96.2)
**Awareness of the rubella outbreak among adults in 2013**								
** **Yes	32	(29.4)	77	(70.6)	15	(4.1)	348	(95.9)
** **No	4	(10.3)	35	(89.7)	8	(1.8)	426	(98.2)
**Awareness of the possibilities of congenital rubella syndrome in case of rubella infection during pregnancy**								
** **Yes	33	(28.4)	83	(71.6)	20	(5.0)	383	(95.0)
** **No	3	(9.4)	29	(90.6)	3	(0.8)	391	(99.2)
**Awareness of the increasing number of congenital rubella syndrome cases**								
** **Yes	27	(33.8)	53	(66.2)	9	(4.7)	182	(95.3)
** **No	9	(13.2)	59	(86.8)	14	(2.3)	592	(97.7)

**Table 5:** Vaccination or antibody testing for rubella in 2013 for women.

	Current desire for pregnancy for herself				Without current desire for pregnancy for herself			
	Vaccinated or tested		Not vaccinated or tested		Vaccinated or tested		Not vaccinated or tested	
	(n = 67)		(n = 208)		(n = 49)		(n = 620)	
**Having acquaintances who have had rubella vaccination**								
** **Yes	18	(58.1)	13	(41.9)	8	(38.1)	13	(61.9)
** **No	49	(20.1)	195	(79.9)	41	(6.3)	607	(93.7)
**Understand the government’s recommendation**								
** **Yes	62	(35.2)	114	(64.8)	43	(13.7)	271	(86.3)
** **No	5	(5.1)	94	(94.9)	6	(1.7)	349	(98.3)
**Being able to confirm rubella vaccination history by the Maternal and Child Health**								
** **Yes	49	(34.0)	95	(66.0)	39	(13.8)	244	(86.2)
** **No	18	(13.7)	113	(86.3)	10	(2.6)	376	(97.4)
**Marital Status**								
** **Single	9	(8.7)	95	(91.3)	11	(4.5)	235	(95.5)
** **Married	57	(34.8)	107	(65.2)	37	(9.6)	348	(90.4)
** **Divorced	1	(14.3)	6	(85.7)	1	(2.6)	37	(97.4)
**Awareness of the rubella outbreak among adults in 2013**								
** **Yes	62	(29.4)	149	(70.6)	47	(10.0)	425	(90.0)
** **No	5	(7.8)	59	(92.2)	2	(1.0)	195	(99.0)
**Awareness of the possibilities of congenital rubella syndrome in case of rubella infection during pregnancy**								
** **Yes	66	(28.3)	167	(71.7)	47	(8.7)	492	(91.3)
** **No	1	(2.4)	41	(97.6)	2	(1.5)	128	(98.5)
**Awareness of the increasing number of congenital rubella syndrome cases**								
** **Yes	49	(35.3)	90	(64.7)	37	(14.9)	212	(85.1)
** **No	18	(13.2)	118	(86.8)	12	(2.9)	408	(97.1)

Tables 6 and 7 present the results of the binomial logistic regression analysis. Compared with participants who had not been tested for rubella antibodies or been vaccinated, the respondents who exhibited rubella prevention behavior had acquaintances who received a rubella vaccination, for men (OR: 2.81; 95% CI: 1.59–3.62) and for women (OR: 2.30; 95% CI: 1.36–3.22) with a current desire for pregnancy, and for men (OR: 8.06; 95% CI: 2.48–18.8) and for women (OR: 3.14; 95% CI: 1.26–6.39) without a current desire for pregnancy. Participants who understood the government recommendation also tended to take action, in both men (OR: 2.65; 95% CI: 1.32–3.60) and women (OR: 2.79; 95% CI: 1.52–3.77) with a current desire for pregnancy, and also men (OR: 5.16; 95% CI: 1.69–13.0) and women (OR: 2.55; 95% CI: 1.05–5.27) without a desire for pregnancy. Similarly, respondents who had been vaccinated or had their antibody levels tested were more likely to be able to confirm their rubella vaccination history using the Maternal and Child Health Handbook in men (OR: 2.22; 95% CI: 1.21–3.16) with a desire for pregnancy, and women (OR: 3.04; 95% CI: 1.61–5.21) without a desire for pregnancy. Married women had significantly higher antibody testing and vaccination rates compared with single or divorced women with (OR: 2.73; 95% CI: 1.71–3.57) or without (OR: 3.00; 95% CI: 1.33–5.76) a current desire for pregnancy. For married men, there was no significant association between being vaccinated among those without a current desire for pregnancy by their partner.

**Table 6:** Statistical associations between vaccination or antibody testing for rubella in 2013 and current desire for pregnancy for partner for men.

	Current desire for pregnancy for partner				Without current desire for pregnancy for partner			
	Crude	95%CI	Adjusted	95%CI	Crude	95%CI	Adjusted	95%CI
**Having acquaintances who have had rubella vaccination**	3.01	(2.03–3.64)	2.81	(1.59–3.62)	11.3	(4.67–20.7)	8.06	(2.48–18.8)
**Understand the government’s recommendation**	2.99	(1.93–3.66)	2.65	(1.32–3.60)	6.19	(2.81–12.1)	5.16	(1.69–13.0)
**Being able to confirm individual rubella vaccination history by the Maternal and Child Health Handbook**	2.89	(2.04–3.50)	2.22	(1.21–3.16)	4.50	(2.07–8.98)	2.50	(0.97–6.03)
**Marital Status**								
** **Single	ref		ref		ref		ref	
** **Married	1.09	(0.56–1.87)	1.07	(0.41–2.16)	2.70	(1.19–5.79)	2.77	(0.96–7.24)
** **Divorced	n.a.		n.a.		2.13	(0.28–12.1)	3.19	(0.35–17.4)
**Awareness of the rubella outbreak among adults in 2013**	1.75	(0.97–2.62)	1.38	(0.38–2.94)	3.38	(1.19–8.57)	0.50	(0.16–1.58)
**Awareness of the possibilities of congenital rubella syndrome which occurs in cases of rubella infection during pregnancy**	2.27	(1.07–3.34)	0.79	(0.17–2.31)	5.83	(1.95–14.1)	2.99	(0.72–10.2)
**Awareness of the increasing number of congenital rubella syndrome cases**	2.13	(1.30–2.93)	1.13	(0.47–2.17)	2.03	(0.89–4.41)	0.53	(0.81–1.55)

CI: Confidence Interval; ref: referent; n.a.: not applicable

**Table 7:** Statistical associations between vaccination or antibody testing for rubella in 2013 and current desire for pregnancy for herself for women.

	Current desire for pregnancy for herself				Without current desire for pregnancy for herself			
	Crude	95%CI	Adjusted	95%CI	Crude	95%CI	Adjusted	95%CI
**Having acquaintances who have had rubella vaccination**	2.74	(1.88–3.46)	2.30	(1.36–3.22)	5.72	(3.01–8.86)	3.14	(1.26–6.39)
**Understand the government’s recommendation**	3.33	(2.38–3.95)	2.79	(1.52–3.77)	5.77	(3.20–8.69)	2.55	(1.05–5.27)
**Being able to confirm individual rubella vaccination history by the Maternal and Child Health Handbook**	2.16	(1.51–2.82)	1.48	(0.88–2.23)	4.40	(2.58–6.73)	3.04	(1.61–5.21)
**Marital Status**								
** **Single	ref		ref		ref		ref	
** **Married	2.76	(1.93–3.46)	2.73	(1.71–3.57)	2.08	(1.12–3.61)	3.00	(1.33–5.76)
** **Divorced	1.50	(0.23–3.68)	1.18	(0.14–3.58)	0.60	(0.08–3.65)	2.33	(0.29–9.06)
**Awareness of the rubella outbreak among adults in 2013**	2.62	(1.57–3.51)	1.10	(0.36–2.43)	6.29	(2.32–10.7)	2.99	(0.67–8.24)
**Awareness of the possibilities of congenital rubella syndrome which occurs in cases of rubella infection during pregnancy**	3.68	(1.73–4.34)	1.05	(0.12–3.43)	4.45	(1.42–9.15)	0.86	(0.16–3.81)
**Awareness of the increasing number of congenital rubella syndrome cases**	2.27	(1.61–2.92)	1.27	(0.69–2.06)	4.36	(2.64–6.55)	2.13	(1.05–3.95)

CI: Confidence Interval; ref: referent

Age and education completed were not statistically associated with any variables in the current study.

## Discussion

In this study we examined potential relationships between social factors of the respondents (20–49 years of age) who took voluntary action and underwent rubella antibody testing or who had been vaccinated against rubella. A current desire for pregnancy was a major factor associated with voluntary action during the Japanese rubella outbreak of 2013, as indicated in Tables 2 and 3. As a result, we undertook additional analysis of the data, as shown in Tables 4–7, to ascertain if they were currently planning to conceive. Few of those without a current desire for pregnancy had voluntarily acted to prevent rubella (3% for men and 7% for women). This result clearly suggests that without a direct perceived benefit, it is difficult to convince individuals to commit financial and time resources to take action which helps prevent infectious diseases. We also identified other social background factors associated with promoting their voluntary action towards rubella protection. These other factors were acquaintances who had been vaccinated, understanding the vaccination recommendations, and confirming their vaccination history by using the Maternal and Child Health Handbook record.

Although financial support for vaccination can be effective to encourage vaccination uptake to a certain extent [13], in the current study it did not appear to be an overly effective method for promoting vaccination (only around one-quarter of respondents had been vaccinated), even among those who expressed a current desire for pregnancy and whose infants were at high risk of congenital rubella syndrome. In compliance with the Japanese government policy, over 1,000 local governments throughout the country had provided partial financial support for rubella vaccination for women with a current desire for pregnancy and / or for the husbands of pregnant women in 2013 [6]. The cost of a dose of measles-rubella vaccine in Japan was approximately 10,000 yen (approximately US$100). The 2013 Japanese rubella outbreak indicated that the 73–90% with positive rubella antibody titer (HI>1:8) proportion in men aged 20–49 was insufficient for protecting the population from this disease [14]. Eliminating rubella through vaccination programs is highly cost-effective at a population level given that the financial burden of congenital rubella syndrome is expensive (up to $140,000 per case in high-income countries) [15].

In the current study, testing rubella antibody levels or vaccination status was statistically associated with having acquaintances who had been vaccinated against rubella. This tendency was especially apparent among men and women who did not express a current desire for pregnancy. The sharing of similar opinions about vaccination in a social network setting is known to have a strong relationship with vaccination rates [16]. Positive attitudes towards rubella vaccination may be amplified among people who share information about the need to be vaccinated. Vaccine accessibility may also be improved among people having acquaintances who take individual action, and are therefore, experienced in the process. These people could then obtain information from their acquaintances regarding the practical steps involved with vaccine-related action.

The results of this study revealed that only 40% of participants understood the 2013 government recommendation for rubella vaccination, even among those who received a specific recommendation to be vaccinated or tested. There is strong evidence that vaccination coverage increases when interventions to enhance access to vaccination services are combined with provider or system-based interventions and / or with interventions to increase client or community demand for vaccination [17]. Barriers to adult immunization include lack of physician recommendations [18] possibly because healthy adults might rarely have the opportunity to be advised by healthcare professionals.

Even though its primary aim is to maintain records during childhood rather than adulthood, the Japanese Maternal and Child Health Handbook nonetheless offers a reliable, hard copy resource that can be used to confirm an individual’s vaccination record in this country [19]. In the current study, participants who determined that they had not been vaccinated tended to get vaccinated or tested. Booster doses of the rubella-containing vaccine did not routinely receive full financial support in Japan until 2006 [4]. An uncertain rubella vaccination history was present in 66% of the men and 56% of the women who were reported rubella cases in 2013 [2]. Systematic maintenance of individual vaccination history records might, therefore, enhance vaccination uptake in the event that additional vaccination is required.

Somewhat surprisingly in the current study, awareness of a rubella outbreak and the possibility of congenital rubella syndrome were not predictors of antibody testing or vaccination, even after adjusting for other study variables. Although it is clearly important to disseminate this kind of information, it may not affect an individual’s behavior to the extent that she or he subsequently takes steps to be vaccinated. Although various positive health behaviors are known to be related in Japan [20], this may not extend to situations where the perception of risk is low [21]. For example, it might be difficult for individuals to comprehend the actual risk of congenital rubella syndrome and how it might affect a baby’s life on a long-term basis, given that it would presumably be quite rare to encounter someone who has been affected by this disease. Nonetheless, it is clearly important to increase awareness regarding the risks of congenital rubella syndrome during rubella outbreaks by using the media, as was achieved during a previous, successful, campaign in Brazil [22].

The current study revealed that marital status among women was a predictor of rubella vaccination. On the contrary for men, no significant association was elucidated between antibody testing or vaccination among those who were married and had a current desire for pregnancy with their partner. Univariate analysis revealed that even for single men with a current desire for pregnancy with their partner, 23.3% had sought testing or vaccination for rubella, a result which was similar to the proportion of married men who had been tested or vaccinated (Table 4). On the contrary, for women, only 8.7% of single women with a current desire for pregnancy had been vaccinated (Table 5), a large gap when compared to those who were married and expressed a current desire for pregnancy (34.8%). As the sample size was relatively small for each category of marital status, further studies will now be needed to ascertain if marital status is a major determinant in taking actions against rubella in Japan, as elsewhere.

It is reasonable to acknowledge that the current study incurred some limitations. Firstly, because a web-based survey was utilized to collect data we cannot ensure that the respondent population represented the general Japanese population. As such, these results, while informative and clearly important, should still be interpreted with caution. Secondly, given that a cross sectional study design was utilized, we cannot confirm that the findings indicate causal relationships. Thirdly, the results of the current study have now revealed some gaps in knowledge which should now be investigated, such as accessibility and barriers to being vaccinated in Japan, as elsewhere.

## Conclusions

Overall, this study suggests that to eliminate rubella outbreaks in Japan, additional initiatives are needed to target those who believe they will, and those who will not, directly benefit from vaccination. Disseminating the government recommendation to all potentially affected subpopulations, along with maintaining life-time vaccination records might offer an additional positive way forwards towards more effective vaccination campaigns in Japan, as elsewhere.
